# Effect of soap bubbles technique, coughing and distraction cards on reducing pain and anxiety during phlebotomy in children

**DOI:** 10.1002/pne2.12090

**Published:** 2022-12-20

**Authors:** Nükhet Balliel

**Affiliations:** ^1^ Public Health Nursing Department, Nursing Faculty Aydın Adnan Menderes University Aydın Turkey

**Keywords:** anxiety, child, health, pain, reducing pain and anxiety

## Abstract

This study aims to compare three techniques (soap bubbles, distraction cards, coughing) to reduce pain and anxiety in children during phlebotomy and collection with the use of a control group. Pain levels of children were assessed with the Wong‐Baker FACES Pain Rating Scale and anxiety levels of children were assessed with the Children's Fear Scale. This randomized controlled study involved intervention groups and a control group. The population of this study consisted of 120 Turkish children (30 subjects in each of the four groups soap bubbles technique, distraction cards, coughing, and control groups) between the ages of 6 and 12. The study found that pain and anxiety levels of the children in intervention groups were lower than those of the children in the control group during phlebotomy procedure and this difference was statistically significant (*P* < .05). No difference was found among the three techniques (Soap bubbles, distraction cards, and coughing) to reduce pain and anxiety in children during the phlebotomy procedure (*P* > .05). Soap bubbles, distraction cards and coughing techniques were found to reduce pain and anxiety in children during phlebotomy. Nurses can play an effective role in reducing pain and anxiety by using these techniques.

## 1 INTRODUCTION

Pain is described as a non‐pleasant emotional and sensory state.^1^, ^2^ A common ground of people is that they all experience pain the first time during their childhood.^3^, ^4^ Pain which is considered as the fifth vital sign should be monitored and managed by healthcare professionals when providing care to pediatric population.^5^, ^6^

Being one of the frightening and acute painful procedures, phlebotomy and venipuncture can cause anxiety in children.^2^, ^7^, ^8^ This painful and fearful experience in childhood can cause afraid and avoid medical procedures during their adult lives. It is recommended to monitor and attempt to reduce pain and anxiety levels of children in simple procedures such as venous blood sampling.^9^ Pain management includes pharmacological and non‐pharmacological approaches.^10^ Various non‐pharmacological approaches can be used to reduce procedural pain in children.^11^, ^12^, ^13^

Researches of both pharmacologic and nonpharmacologic methods in interventional pain relief decisived that nonpharmacologic methods were as effective as pharmacologic methods.^6^, ^8^, ^11^, ^12^ Nonpharmacologic methods are preferred in relieving interventional pain because pharmacologic methods have many side effects. Many studies support the effectiveness of nonpharmacologic methods in managing the pain associated with invasive procedures in children.^6^, ^8^, ^11^, ^12^, ^14^ Non‐pharmacological methods were reported to be effective in reducing pain and fear in children.^15^, ^16^ Non‐pharmacological methods can be classified into three groups: supportive, physical and cognitive and behavioral.^6^, ^8^, ^11^, ^12^ Supportive methods include reading books, watching videos, and having the child's family available during the painful procedure.^8^, ^14^ Physical methods include touching, positioning, massage, skin stimulation, and hot and cold application.^4^, ^8^ Cognitive and behavioral methods include relaxing and distraction techniques.^4^, ^6^

There are two types of distraction techniques; namely active and passive distraction techniques.^6^, ^11^ Active distraction techniques involve children in the process to distract their attention during painful medical procedures. Techniques used for this methods involve using interactive toys, guided images and relaxing, controlled breathing, electronic games, and blowing up balloons.^17^

Distraction techniques are simple and effective methods to distract children's attention away from painful stimulus to reduce anxiety.^4^, ^11^ Attracting attention to more pleasant stimulus can considerably reduce anxiety, fear, and pain.^18^ Furthermore, it also has a positive effect on the mood leading to the release of anti‐stress hormones and eventually mental relaxation.^15^ Distraction is a method widely used by parents, and it can be used by healthcare professionals to reduce pain and anxiety during painful medical procedures.^19^

The studies in the literature reported that distraction cards^3^, ^13^, ^18^, ^19^, ^20^, ^21^ and soap bubbles technique^15^, ^16^, ^22^, ^23^, ^24^ coughing^6^, ^25^, ^26^ technique were effective in reducing pain and anxiety in children during medical procedures. According to the neurocognitive attention model, distraction techniques are believed to be successful in reducing pain and anxiety. The reason for this is that pain is reduced by offering a strong pleasant stimulus to replace pain perception.^18^ Blowing soap bubbles, distraction cards, and coughing were proved to be a successful distraction technique.^16^, ^23^, ^27^, ^28^ Blowing soap bubbles technique was used to reduce pain, anxiety, and fear in children who were waiting to be examined in an emergency room.^15^ Previously published studies have reported that distraction cards were found to be the most effective method for pain and anxiety relief for children during phlebotomy.^3^, ^18^, ^19^ Coughing increases intrathoracic pressure and stimulation to the autonomic nervous system, causing an increase in heart rate and blood pressure, a higher level of pressure in the subarachnoid space, and baroreceptor activation. Coughing was increased in pressure in the subarachnoid space activates the segmental pain‐inhibiting pathways; thus, the increase in blood pressure and baroreceptor activation appears to be efficacious in reducing the perception of pain in these studies.^6^, ^25^, ^26^

### 1.1 Study purpose

The aim of this study is to determine comparative effect of blowing soap bubbles, distraction cards, and coughing to reduce pain, fear, and anxiety in children during phlebotomy.

## 2 METHODS

### 2.1 Study design

This was an experimental, randomized controlled study.

### 2.2 Setting and sample

The population of this study consisted of children between the ages of 6 to 12 in the phlebotomy department of an obstetrics, gynecology, and children's hospital in Turkey. Healthy children between the ages 6 and 12 who needed blood tests were included in the study. Children who had neurological development disorders, who could not have verbal communication, who were hearing or visually impaired, who had taken analgesic agents minimum 6 hours before phlebotomy procedure, and who had a history of fainting during phlebotomy were excluded from the study.

Minimum sample size is set as 30 in situations where intervention and control groups are used in the experimental studies. None of these children had a history of using analgesics for at least 6 hours. Therefore, 120 children who met the inclusion criteria were included in the study for the three intervention groups and the control group.

In the analysis done with Gpower 3.1 software program, when the effect size (w) was 0.68, 1−*β* = 0.80 and *α* = 0.05, the number of people that should be included in each group was found to be 28.^20^ 120 children were included in the study and they were assigned to four groups; distraction cards (n = 30), soap bubbles (n = 30), coughing (n = 30), and control group (n = 30).

### 2.3 Ethical considerations

The approval of the Ethics Board of a university in Turkey was obtained for this study. Written consents from the children and families were obtained after providing them information about the study.

### 2.4 Data collection

A sociodemographic form was filled out for each child who agreed to participate in the study for randomization purposes. The researcher provided information about the study and about the scoring in Wong‐Baker FACES Pain Rating Scale and Children's Fear Scale to the children and their parents. The same person used the same size needle to draw blood from each children. Data were collected with face‐to‐face interviews.

### 2.5 Procedure

Venipuncture was performed between 08:00‐12:00 hours and 13:00‐16:00 hours with a vacutainer and a 21‐gauge needle. No topical anesthetic was used as it is not the standard practice of the unit. After the procedure, the children's pain levels were assessed by self‐report and parent's and the observer's report

### 2.6 Randomization

**FIGURE 1:**
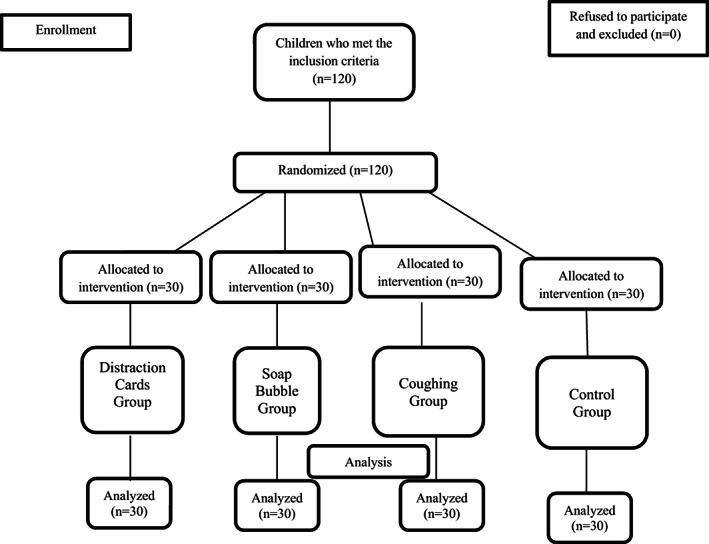
Diagram showing the flow of participants

Children (N = 120) were then assessed according to the inclusion criteria and invited to participate depending on their eligibility. A computer‐based random number generator was used to assign the patients into groups. Children who met the inclusion criteria were randomly selected from the list of names and assigned to groups. The flow chart for this randomized controlled study was shown in Figure 1.

### 2.7 Instruments

#### 2.7.1 Child sociodemographic form

This form includes 4 questions about the gender, age, weight, and height to calculate body mass index.

#### 2.7.2 Wong‐Baker FACES pain rating scale

The original of the scale was developed by Donna Wong and Connie Morain Baker in 1981 to assess pain in children (between the ages of 3 and 18) and the scale was revised in 1983. It is a scale of 0‐10. “0” means that the child does not have any pain and is shown with a happy smiling face and “10” point means that the child's pain is very severe and is shown with a crying face. The pain level of the child can be determined with the Wong‐Baker FACES Pain Rating Scale based on self‐reporting of the child and the reports of the parent and observer.

#### 2.7.3 Children's fear scale

This scale was used to determine anxiety levels of children during the procedure for blood taking according to their facial expressions. It uses a scale of 0‐4. It consists of 5 facial expressions. Scores are given according to each facial expression. A face picture with no reaction shows that the child is not afraid (0 point), a frightened face picture means that the child is very afraid (4 points).^29^

#### 2.7.4 Distraction cards

Distraction cards were visual cards with the size of (Flippits1, MMJ Labs, Atlanta, Georgia, USA) 5 × 8 cm^2^, each of which include a different picture and shape. Since the native language of the children included in the study was not English, a bilingual expert translated the cards. Children first observed the card shown carefully and then the researcher asked them to answer the questions about the card. The children were asked several questions about the visuals on the cards such as “Can you see the butterfly in the picture?”, “How many dogs are there in the picture?”, “Are there any birds in this picture?”. Distraction cards were started to be used just before the phlebotomy procedure began and continued after the procedure was completed.

#### 2.7.5 Soap bubbles

Soap bubbles technique was used as the distraction method. A soap bubble kit which was sealed and had not been opened was used. Children in the experimental group were involved in an activity in which researcher blew big soap bubbles (about 50 to 100 cm) and asked children to interact with them by looking at, blowing, and/or popping the bubbles. The activity lasted for about 15 min.

#### 2.7.6 Coughing

Each child was shown by the researcher the way they should be coughing before phlebotomy. The procedure was not started until the child learned to cough in the right way. Then, the child was asked to inhale before the needle was inserted to draw blood and to cough during the procedure. Children in the cough trick group were told that they would be asked to cough while their blood was being taken. As a preliminary exercise, the children were asked before the procedure to take a deep breath and then cough actively.

#### 2.7.7 Control group

No intervention was used to reduce pain or anxiety in the children in this group during phlebotomy.

### 2.8 Data analysis

Statistical Package for the Social Sciences (SPSS Inc., Chicago, IL, USA) 21.0 for windows was used for statistical analysis. Descriptive statistics such as numbers, percentage, and parametric data were analyzed with the chi square test. Independent groups were evaluated with one‐way variance analysis and *t*‐test. Bonferroni test was used for post‐hoc analysis. Statistical significance level of <.05 was used.

## 3 RESULTS

The mean age of the children included in the study was 8.76 ± 1.28 (min‐max age:6‐12). Age and body mass index (BMI) were similar in all four groups (Table 1). No significant difference in anxiety levels of children was found between groups before phlebotomy (*P* > .05).

**TABLE 1:** Baseline characteristics and pre‐procedural anxiety levels of the four study groups.

	Soap bubbles group (n = 30) Mean ± *SD*	Distraction cards group (n = 30) Mean ± *SD*	Coughing group (n = 30) Mean ± *SD*	Control group (n = 30) Mean ± *SD*	*F*	*P*
Age	8.42 ± 2.91	8.82 ± 1.23	8.93 ± 1.24	8.85 ± 2.41	0.582	.832
BMI	15.43 ± 3.21	17.32 ± 4.32	16.28 ± 2.54	15.63 ± 3.21	1.821	.471
Pre‐procedural anxiety levels						
Self‐reported	1.67 ± 1.15	1.23 ± 1.25	1.30 ± 1.29	2.05 ± 0.74	1.851	.065
Parent reported	1.73 ± 2.46	1.20 ± 1.46	1.51 ± 1.34	2.03 ± 1.24	2.368	.824
Observer reported	1.66 ± 1.25	1.90 ± 1.48	1.43 ± 1.44	2.14 ± 1.32	2.086	.108

### 3.1 Comparison of the groups for pain levels

Pre‐procedural and procedural pain levels in each group were shown in Table 2. There was no significant difference in pain levels of children in the groups before the phlebotomy procedure (*P* > .05). According to the self‐reports of children and reports of their parents and the researcher, the intervention group which had the least pain was the soap bubbles group; however, there was no significant difference between three intervention groups during phlebotomy (*P* > .05). However, a statistically significant difference was found between the intervention groups and the control group during phlebotomy (*P* < .05). Accordingly, the pain level of the control group was higher than the intervention groups during the procedure (*P* = .002).

**TABLE 2:** Comparison of the groups for pain levels.

	Soap bubbles group (n = 30)	Distraction cards group (n = 30)	Coughing group (n = 30)	Control group (n = 30)	*F*	*P*
Pre‐procedural pain scores						
Self‐reported	0.22 ± 0.56	0.25 ± 0.37	0.18 ± 0.77	0.36 ± 0.88	0.963	.832
Parent reported	0.22 ± 0.56	0.25 ± 0.37	0.18 ± 0.77	0.36 ± 0.88	0.963	.832
Observer reported	0.22 ± 0.56	0.25 ± 0.37	0.18 ± 0.77	0.36 ± 0.88	0.963	.832
Procedural pain scores						
Self‐reported	1.24 ± 2.21	1.76 ± 1.26	1.32 ± 1.25	3.58 ± 1.41	0.521	.002
Parent reported	1.15 ± 1.95	1.68 ± 0.36	1.26 ± 2.28	3.45 ± 1.02	0.458	.002
Observer reported	1.12 ± 1.86	1.52 ± 1.78	1.21 ± 1.53	3.40 ± 1.06	1.231	.002

There was no significant difference in anxiety levels of children in the intervention groups during the procedure for phlebotomy based on reporting of parents and the researcher (*P* > .05). The anxiety level of the control group was significantly higher than the intervention group during the procedure (*P* = .000) (Table 3).

**TABLE 3:** Comparison of the Groups for Anxiety Levels.

	Soap bubbles group (n = 30)	Distraction cards group (n = 30)	Coughing group (n = 30)	Control group (n = 30)	*F*	*P*
Procedural anxiety levels						
Self‐reported	0.86 ± 0.63	1.65 ± 1.27	0.96 ± 0.54	3.78 ± 0.53	1.253	.000
Parent reported	0.92 ± 0.35	1.70 ± 1.53	0.98 ± 0.37	3.89 ± 0.28	0.352	.000
Observer reported	0.72 ± 0.48	1.43 ± 1.64	0.75 ± 0.28	3.92 ± 0.64	0.245	.000

## 4 DISCUSSION

Medical procedures can be painful and cause anxiety for children in hospitals. The American Society for Pain Management Nursing recommends using pharmacological and non‐pharmacological approaches to manage pain before and during painful medical procedures. The effect of distraction technique which is one of the non‐pharmacological methods to reduce pain can be explained with the fact that the brain focuses on distracting stimulus reducing the feel of pain as its capacity to concentrate attention is limited.

There are studies in the literature, which report that various distraction methods are effective to reduce pre‐procedural and procedural pain and anxiety in children.^3^, ^13^, ^30^, ^31^, ^32^, ^33^ Similar to the literature; this study also reported that distractive methods are effective in reducing pain and anxiety in children during phlebotomy.

There are limited number of studies which use soap bubbles technique to reduce pain and anxiety in children.^15^, ^16^, ^23^, ^27^ The use of soap bubbles technique before and after a medical procedure was reported to be effective in reducing pain in children.^16^, ^23^, ^27^ In their study, Longobardi et al used soap bubbles technique not during medical procedures but for children waiting in the pediatric emergency room before and after examination to reduce pain and anxiety and reported that the technique was effective.^15^ Similar to previous studies, the soap bubbles technique which is cheap and easy to use was determined to have a pain and anxiety reducing effect during medical procedures in children. The soup bubbles group had lower level of pain and anxiety than the control group and the difference was found to be significant. Although the pain and anxiety level of the soap bubbles group was lower than other intervention groups, this difference was not significant. Soap bubbles technique is recommended as it can distract children which is easy and cheap.

Another method used to reduce pain and anxiety in children during medical procedures is the distraction cards method. Recent studies have demonstrated that distracting cards are very effective in reducing pain and anxiety in children during medical procedures.^13^, ^20^, ^31^, ^32^, ^33^ Similar to the literature, this study showed that distraction cards reduced pain and anxiety during phlebotomy and the children in the distraction cards group had significantly lower level of pain and anxiety than those in the control group. As distraction cards are visually appealing and require asking questions to children allowing them to talk and are easy to use, they are recommended for using to reduce pre‐procedure and procedural pain and anxiety.

There are fewer studies on coughing, one of the techniques used to reduce pain and anxiety during medical procedures in children.^6^, ^25^, ^26^ This study showed that pain and anxiety level in coughing group was significantly lower than the control group. However, the difference between the pain and anxiety levels of the coughing group and the other intervention groups (soap bubbles technique and distraction cards) was not significant. A study which used coughing to reduce pain and anxiety in adult patients during medical procedure reported that this technique was effective.^34^ Unlike the other studies, this study used and compared three different techniques; soap bubbles, distraction cards, and coughing in groups. A previously study reported that coughing was more successful in reducing pain and fear compared to other techniques but the difference was not significant.^26^ A study conducted with adult groups, which support our study findings reported that coughing technique created a significant difference in reducing pain during phlebotomy.^34^ Different from our findings in the study, the study conducted with children determined that using coughing technique during vaccination shots did not create a significant difference.^25^ The results are thought to be caused by the fact that techniques used and compared in studies are different; study groups consist of people of different age groups and techniques are applied by people with varying skills. Although the number of studies which use coughing technique is limited, this technique is thought to be an effective method to reduce pain and anxiety.

### 4.1 Limitations

Limitations of this study; this is not a double‐blind study as the researcher knew the group which every child was assigned to. Assessment of pain and anxiety was based on self‐reporting and researcher could be more biased when assessing pain and anxiety before and after the procedure depending on the reports of the parents and the child.

## 5 CONCLUSIONS

It is important that nurses assess medical pre‐procedural and procedural pain. Pain management is one of the independent roles of nurses and it is a factor which improves the quality of healthcare they provide; therefore, it is a responsibility nurses should consider to take. Although soap bubbles technique was the most effective method in this study, there was no difference between soap bubbles technique and distraction cards and coughing techniques in reducing pain and anxiety. However, there is a significant difference in the pain and anxiety level between all of the intervention groups and the control group. Nurses can play an effective role in reducing pain and anxiety by using these techniques. Further studies should aim to produce more evidence for the use of non‐pharmacological methods for pain management. Nurses working in clinics and on the field should develop guidelines which they can use in practice and present the results in the literature.
